# Supplementary material for: Mechanosensitive TRPV4 immunohistochemistry improves deep learning‐based classification of ductal carcinoma in situ beyond H&E morphology
Source: J Pathol Clin Res. 2026 Jul 8;12(4):e70106. doi: 10.1002/2056-4538.70106 (PMC13343292; doi:10.1002/2056-4538.70106)
Supplement: Supplementary file 1 — Table S1. Cohort characteristics and tile‐level dataset composition Table S2. Summary of deep learning architecture, training, and inference settings Table S3. Internal cross‐validation performance (UVA cohort) Table S4. Per‐class internal cross‐validation performance (UVA cohort) Table S5. Patient‐level per‐class performance on GWU test cohort Figure S1. Representative 299 × 299 input tiles for each diagnostic group Figure S2. Tile‐level error structure on the GWU external test cohort [file CJP2-12-e70106-s001.pdf]

Mechanosensitive TRPV4 immunohistochemistry improves deep learning-based classification of ductal carcinoma *in situ* beyond H&E morphology

J Yoo, R Karthikeyan *et al*. *J Pathol Clin Res* https://doi.org/10.1002/2056-4538.70106

Supplementary Tables S1–S5

Supplementary Figures S1–S2

Characteristic	UVA (Development)	GWU (External Test)	Total
**Unique patients**	69	39	**108**
Patients with H&E	62	31	93
Patients with TRPV4 IHC	51	33	84
Patients with both modalities	44	25	69
**Total tiles (after intermediate-grade DCIS exclusion)**	16,415	7,833	**24,248**
**H&E tiles**	**9,623 (58.6%)**	**4,511 (57.6%)**	**14,134 (58.3%)**
Normal/benign	1,367 (14.2%)	1,454 (32.2%)	2,821 (20.0%)
ADH/low-grade DCIS	2,878 (29.9%)	474 (10.5%)	3,352 (23.7%)
High-grade DCIS	3,252 (33.8%)	1,786 (39.6%)	5,038 (35.6%)
IDC	2,126 (22.1%)	797 (17.7%)	2,923 (20.7%)
**TRPV4 IHC tiles**	**6,792 (41.4%)**	**3,322 (42.4%)**	**10,114 (41.7%)**
Normal/benign	1,338 (19.7%)	770 (23.2%)	2,108 (20.8%)
ADH/low-grade DCIS	1,600 (23.6%)	495 (14.9%)	2,095 (20.7%)
High-grade DCIS	1,534 (22.6%)	1,480 (44.5%)	3,014 (29.8%)
IDC	2,320 (34.2%)	577 (17.4%)	2,897 (28.6%)
**Cross-validation folds (UVA only)**			
H&E: Fold sizes (patients)	22, 21, 19	—	—
IHC: Fold sizes (patients)	19, 16, 16	—	—

Table S1. Cohort characteristics and tile-level dataset composition. Patient- and tile-level characteristics for the UVA development cohort and GWU external test cohort after excluding intermediate-grade DCIS cases. Overall, 108 unique patients contributed 24,248 tiles across H&E and TRPV4 IHC. Modality availability varied by patient due to tissue exhaustion and/or quality-control exclusions; therefore, per-modality patient counts and fold sizes differ (UVA). The UVA cohort was used for patient-level three-fold cross-validation, and the GWU cohort served as a fully held-out external test set for all models. Class distributions reflect real-world diagnostic case mix, with normal/benign and ADH/low-grade DCIS under-represented relative to high-grade DCIS. Percentages in parentheses denote the proportion within each institution and modality.

Parameter	Setting
Task	4-class classification (normal/benign, ADH/LG-DCIS, HG-DCIS, IDC)
Modalities	H&E and TRPV4 IHC
Development cohort	UVA; patient-level 3-fold cross-validation
External test cohort	GWU; fully held out
Input	Non-overlapping ROI-derived image tiles
Tile size	299 × 299 pixels
Backbones	Xception; EfficientNet-B0
Initialization	ImageNet pretrained
Classification head	Global average pooling, Dense (256, ReLU, L2 = 0.001), Dropout (0.6), and then 4-class softmax
Transfer learning	Two-stage training: frozen backbone, then fine-tuning
Optimizer	Adam
Loss	Class-weighted, label-smoothed categorical cross-entropy
Class imbalance handling	Inverse-frequency class weighting with additional IDC upweighting
Alternative loss explored	Class-weighted focal loss (not used for primary analyses)
Augmentation	Random flips, 90° rotations, zoom/cropping, color jitter, Gaussian noise
Precision policy	Mixed precision (mixed-float16); final layer in float32
Batch size	4
Software	TensorFlow 2.10.1/Keras
Hardware	NVIDIA GeForce RTX 4090 GPU
Approximate training time	6-17 min per model per fold
Ensemble inference	Mean probability across 3 fold-specific models
Patient-level prediction	Mean probability pooling across tiles
Evaluation levels	Tile level and patient level
Main summary metrics	Macro-F1 and macro-AUC
Uncertainty estimation	Non-parametric bootstrap, 500 resamples
Error analysis	Adjacent-grade vs non-adjacent misclassification

Table S2. Summary of deep learning architecture, training, and inference settings. Principal deep learning settings used for H&E and TRPV4 IHC classification. Models were trained on the UVA development cohort using patient-level 3-fold cross-validation and evaluated on the fully held-out GWU external test cohort without retraining or fine-tuning.

Architecture	Modality	Fold	Tile-level Macro-F1	Tile-level Macro-AUC	Patient-level Macro-F1	Patient-level Macro-AUC	n patients
**EfficientNet-B0**	**H&E**	0	0.59	0.89	0.60	0.89	22
		1	0.36	0.71	0.51	0.73	21
		**2**	**0.74**	**0.92**	**0.69**	**0.90**	19
		Mean± SD	0.56± 0.19	0.84± 0.11	0.60± 0.09	0.84± 0.10	—
	**TRPV4 IHC**	0	0.49	0.83	0.64	0.95	19
		**1**	**0.65**	**0.90**	**0.84**	**0.98**	16
		2	0.60	0.88	0.79	0.98	16
		Mean± SD	0.58± 0.08	0.87± 0.03	0.76± 0.11	0.97± 0.02	—
**Xception**	**H&E**	0	0.57	0.83	0.64	0.93	22
		1	0.51	0.80	0.49	0.81	21
		**2**	**0.69**	**0.93**	**0.69**	**0.93**	19
		Mean± SD	0.59± 0.09	0.85± 0.07	0.61± 0.10	0.89± 0.07	—
	**TRPV4 IHC**	**0**	**0.64**	**0.90**	**0.84**	**0.98**	19
		1	0.76	0.94	0.66	0.95	16
		2	0.64	0.87	0.74	0.97	16
		Mean± SD	0.68± 0.07	0.90± 0.04	0.75± 0.09	0.97± 0.02	—

Table S3. Internal cross-validation performance (UVA cohort). Detailed three-fold, patient-stratified cross-validation results on the UVA development cohort. Each row reports performance on the held-out validation fold. For each architecture–modality combination, the fold with the best patient-level performance is highlighted in bold (based on patient-level macro-F1, with patient-level macro-AUC as a tie-breaker when needed). For external testing, predictions are generated by ensembling the three fold-specific models per modality and architecture (see Table 1). Fold sizes vary due to patient-level stratification. SD = standard deviation across three folds.

H&E-EfficientNet-B0

Class	Sensitivity (mean ± SD)	Precision (mean ± SD)	F1-score (mean ± SD)	VAL tiles (fold0/fold1/fold2)
Normal/Benign	0.90 ± 0.06	0.44 ± 0.13	0.59 ± 0.12	591/397/379
ADH/LG-DCIS	0.70 ± 0.10	0.62 ± 0.21	0.65 ± 0.16	1144/984/750
HG-DCIS	0.45 ± 0.34	0.59 ± 0.30	0.51 ± 0.33	1560/856/836
IDC	0.40 ± 0.25	0.86 ± 0.13	0.50 ± 0.27	1012/731/383

TRPV4 IHC-EfficientNet-B0

Class	Sensitivity (mean ± SD)	Precision (mean ± SD)	F1-score (mean ± SD)	VAL tiles (fold0/fold1/fold2)
Normal/Benign	0.84 ± 0.11	0.48 ± 0.10	0.60 ± 0.07	734/322/282
ADH/LG-DCIS	0.76 ± 0.20	0.60 ± 0.17	0.67 ± 0.17	645/501/454
HG-DCIS	0.51 ± 0.12	0.65 ± 0.26	0.56 ± 0.16	658/503/373
IDC	0.37 ± 0.28	0.94 ± 0.09	0.50 ± 0.27	1184/686/450

H&E-Xception

Class	Sensitivity (mean ± SD)	Precision (mean ± SD)	F1-score (mean ± SD)	VAL tiles (fold0/fold1/fold2)
Normal/Benign	0.81 ± 0.05	0.48 ± 0.12	0.59 ± 0.09	591/397/379
ADH/LG-DCIS	0.61 ± 0.01	0.66 ± 0.24	0.62 ± 0.12	1144/984/750
HG-DCIS	0.58 ± 0.29	0.63 ± 0.08	0.58 ± 0.19	1560/856/836
IDC	0.49 ± 0.28	0.76 ± 0.07	0.57 ± 0.24	1012/731/383

TRPV4 IHC-Xception

Class	Sensitivity (mean ± SD)	Precision (mean ± SD)	F1-score (mean ± SD)	VAL tiles (fold0/fold1/fold2)
Normal/Benign	0.91 ± 0.07	0.52 ± 0.12	0.65 ± 0.09	734/322/282
ADH/LG-DCIS	0.67 ± 0.17	0.80 ± 0.13	0.71 ± 0.10	645/501/454
HG-DCIS	0.69 ± 0.18	0.74 ± 0.19	0.69 ± 0.08	658/503/373
IDC	0.57 ± 0.37	0.93 ± 0.09	0.67 ± 0.29	1184/686/450

Table S4. Per-class internal cross-validation performance (UVA cohort). Per-class sensitivity, precision, and F1 score are computed on validation tiles for each held-out fold of patient-stratified 3-fold cross-validation. Values are reported as mean ± SD across three folds. The final column lists the number of validation tiles per fold (fold0/fold1/fold2).

Model	Class	Sensitivity	Specificity	Precision	F1 [95% CI]	AUC [95% CI]	n patients
**H&E EfficientNet-B0**	Normal/benign	0.93	0.50	0.64	0.76 [0.56–0.89]	0.91 [0.80–0.99]	15
	ADH/LG-DCIS	0.25	0.93	0.33	0.29 [0.00–0.67]	0.70 [0.41–0.97]	4
	HG-DCIS	0.40	0.92	0.50	0.44 [0.00–0.83]	0.91 [0.77–1.00]	5
	IDC	0.14	0.96	0.50	0.22 [0.00–0.60]	0.69 [0.48–0.87]	7
**IHC EfficientNet-B0**	Normal/benign	0.93	0.79	0.77	0.84 [0.67–0.97]	0.95 [0.85–1.00]	14
	ADH/LG-DCIS	1.00	0.93	0.67	0.80 [0.33–1.00]	0.95 [0.82–1.00]	4
	HG-DCIS	0.86	0.96	0.86	0.86 [0.55–1.00]	0.98 [0.92–1.00]	7
	IDC	0.25	0.96	0.67	0.36 [0.00–0.71]	0.77 [0.58–0.96]	8
**H&E Xception**	Normal/benign	0.47	0.94	0.88	0.61 [0.35–0.82]	0.84 [0.68–0.97]	15
	ADH/LG-DCIS	0.50	0.78	0.25	0.33 [0.00–0.67]	0.61 [0.24–1.00]	4
	HG-DCIS	1.00	0.77	0.46	0.63 [0.31–0.86]	0.85 [0.70–0.98]	5
	IDC	0.14	0.88	0.25	0.18 [0.00–0.50]	0.61 [0.38–0.82]	7
**IHC Xception**	Normal/benign	0.93	0.79	0.77	0.84 [0.69–0.96]	0.90 [0.76–1.00]	14
	ADH/LG-DCIS	0.25	1.00	1.00	0.40 [0.00–1.00]	0.94 [0.80–1.00]	4
	HG-DCIS	0.86	0.92	0.75	0.80 [0.55–1.00]	0.99 [0.95–1.00]	7
	IDC	0.63	0.92	0.71	0.67 [0.32–0.91]	0.85 [0.69–0.98]	8

Table S5. Patient-level per-class performance on GWU test cohort. Patient-level per-class performance on the GWU external test cohort. Patient-level predictions were obtained by mean-probability pooling across all tiles from each patient. Per-class sensitivity, specificity, precision, F1 score, and AUC are reported with 95% confidence intervals estimated by bootstrap resampling (n = 500). Sensitivity, specificity, and AUC are prevalence-independent measures, whereas precision and F1 score are prevalence-dependent and should be interpreted in light of class support.

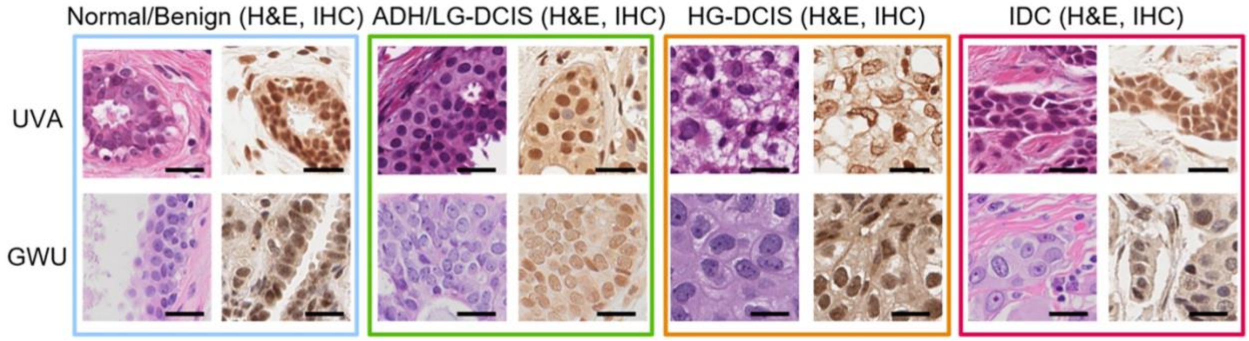

Figure S1. Representative 299×299 input tiles for each diagnostic group. For each of the four ordered diagnostic groups, normal/benign (blue box), ADH/low-grade DCIS (ADH/LG-DCIS; green box), high-grade DCIS (HG-DCIS; orange box), and invasive ductal carcinoma (IDC; red box), representative tiles from the UVA (top row) and GWU (bottom row) cohorts are shown, with paired H&E (left) and TRPV4 IHC (right) tiles for each cohort. Tiles illustrate the local morphology and staining patterns used as input to the convolutional neural networks, at the same resolution and field of view as during model training and evaluation. Examples are intended to highlight typical appearances within each group rather than to capture the full spectrum of histologic variability. Scale bar, 40 µm.

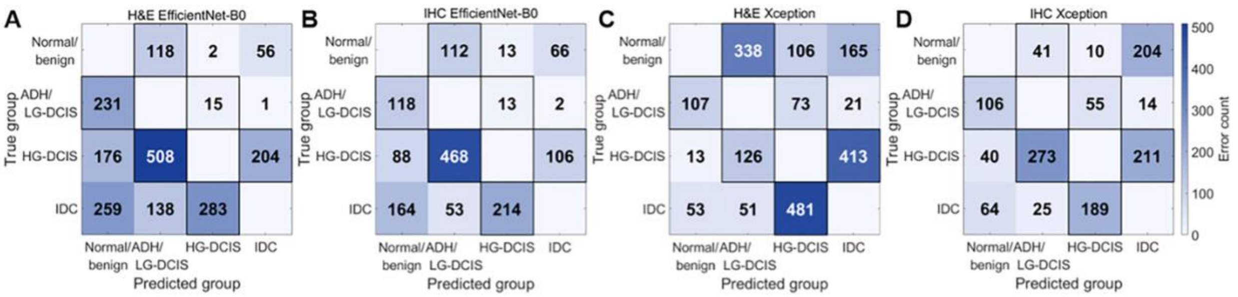

Figure S2. Tile-level error structure on the GWU external test cohort. Heatmaps show misclassified tiles (diagonal entries set to zero) for the four models evaluated on the GWU test set. **(A)** H&E EfficientNet-B0; **(B)** TRPV4 IHC EfficientNet-B0; **(C)** H&E Xception; **(D)** TRPV4 IHC Xception. Color intensity encodes the error count in each true-predicted class cell; gray boxed cells highlight adjacent-grade errors (normal/benign–ADH/LG-DCIS, ADH/LG-DCIS–HG-DCIS, HG-DCIS–IDC). For panels **A–D**, adjacent-grade errors account for 68.3% (1359/1991), 72.8% (1031/1417), 79.0% (1538/1947), and 71.0% (875/1232) of all misclassified tiles, respectively.
